# Effects of Deep Transcranial Magnetic Stimulation on Cognitive Function in Bipolar Depression: A Randomized Controlled Trial Using the MATRICS Consensus Cognitive Battery

**DOI:** 10.31083/AP47409

**Published:** 2026-02-28

**Authors:** Lijun Chu, Xiaoju Jia, Ping Gao, Xia Sun, Jian Zhang, Yu Ding, Shiwang Chen, Fuyou Bi, Chuhao Zhang, Dazhi Li, Yong Zhang

**Affiliations:** ^1^Unit of Bipolar Disorder, Tianjin Anding Hospital, Mental Health Center of Tianjin Medical University, 300222 Tianjin, China; ^2^Institute of Mental Health, Tianjin Anding Hospital, Mental Health Center of Tianjin Medical University, 300222 Tianjin, China

**Keywords:** bipolar disorder, transcranial magnetic stimulation, cognitive dysfunction, depression, randomized controlled trial

## Abstract

**Background:**

Bipolar disorder (BD) is characterized by persistent cognitive deficits. These deficits contribute to functional impairment and often respond poorly to pharmacotherapy. Although deep transcranial magnetic stimulation (dTMS) has demonstrated antidepressant efficacy, there is limited knowledge about its cognitive effects and comprehensive clinical performance in BD. In this study, we assessed the cognitive outcomes, clinical efficacy, and safety of H1-coil dTMS in BD patients.

**Methods:**

In this randomized, double-blind, sham-controlled trial, 100 inpatients with BD received 4 weeks of active or sham H1-coil dTMS. The MATRICS (Measurement and Treatment Research to Improve Cognition in Schizophrenia) Consensus Cognitive Battery (MCCB) was used to investigate the cognitive function, and the 17-item Hamilton Depression Rating Scale (HDRS-17) was used to assess depressive symptoms from baseline to week 4.

**Results:**

Both groups, active and sham dTMS, showed significant cognitive improvements across most domains (*p* < 0.05), with no statistically significant between-group differences (all *p *> 0.05). At the endpoint, the active dTMS group showed statistically significantly lower HDRS-17 scores and a higher response rate than the sham group (mean difference = 2.94, 95% CI [0.10, 5.78], *p* = 0.04); 50% vs. 24%; OR = 3.17, 95% CI [1.35, 7.44], *p* = 0.007). All treatments demonstrated a favorable safety profile, with only mild and transient adverse effects.

**Conclusions:**

In patients with BD, active dTMS was well-tolerated and was associated with a higher response rate and statistically significant (albeit modest) lower depressive symptom scores compared to sham stimulation, without inducing cognitive adverse effects. However, no specific cognitive benefit beyond its antidepressant effect was established. Overall, these results indicate that dTMS has potential as an adjunctive treatment option for bipolar depression, particularly when medications are limited or poorly tolerated.

**Clinical Trial Registration:**

NCT06524505. Registered 23 July, 2024, https://clinicaltrials.gov/study/NCT06524505.

## Main Points

1. Active H1-coil deep transcranial magnetic stimulation (dTMS) did not confer specific cognitive benefits in bipolar depression. Although cognitive function improved in both active and sham dTMS groups, no significant between-group difference was observed.

2. dTMS produced a modest but statistically significant reduction in depressive symptom scores and more than twice the response rate (50% vs. 24%) compared to sham treatment. 

3. dTMS demonstrated a favorable safety and tolerability profile, with only mild and transient adverse events reported. 

4. These results position dTMS as a potential adjunctive treatment option for bipolar depression, particularly when standard pharmacotherapy is ineffective or poorly tolerated.

## 1. Introduction

Bipolar disorder (BD) is a lifelong psychopathological condition characterized by cyclical alternating phases of mania and depression, with a global prevalence of approximately 2–3% [1]. In addition to marked mood fluctuations, persistent cognitive impairments and social dysfunction are recognized as core features of the disorder. These deficits significantly affect patients’ quality of life and social adaptability, ranking BD among the most disabling mental disorders globally.

Depressive episodes represent the predominant and most disabling phase of BD. They frequently involve widespread cognitive deficits affecting attention, executive function, memory, and processing speed [2, 3, 4, 5, 6]. These impairments often persist during euthymia. Meta-analyses confirm significant cognitive impairment in BD compared to healthy populations [7, 8, 9]. Cognitive impairment is now recognized as a fundamental characteristic of BD and predicts poor psychosocial and occupational outcomes [10, 11]. Cognitive dysfunction in BD includes both trait-like impairments that persist during euthymia and state-dependent deficits that fluctuate with mood episodes. Trait-related deficits, particularly in executive control and working memory, are thought to reflect enduring abnormalities in prefrontal–limbic networks [12]. In contrast, state-related impairments may improve as mood symptoms remit [13, 14]. Although pharmacological agents (e.g., lithium, valproate, and antipsychotics) effectively stabilize mood, their ability to improve cognitive function remains limited. Furthermore, these medications may cause adverse effects such as psychomotor slowing and memory impairment [15, 16]. Therefore, non-pharmacological neuromodulation approaches aimed at enhancing cognitive function in BD have become a major research focus. Besides, treatment-resistant bipolar depression (TRBD) remains a significant clinical issue, as many patients fail to achieve adequate remission despite optimized pharmacotherapy or augmentation strategies [17]. This unmet clinical need underscores the importance of exploring safe and effective non-pharmacological interventions, such as deep transcranial magnetic stimulation (dTMS).

Repetitive transcranial magnetic stimulation (rTMS) is a noninvasive neuromodulation technique. The dorsolateral prefrontal cortex (DLPFC) is commonly targeted, given its crucial role in emotional regulation [18]. Transcranial magnetic stimulation (TMS) received the U.S. Food and Drug Administration (FDA) designation as a Breakthrough Device in 2020 for the treatment of BD. Moreover, meta-analyses have demonstrated significantly higher response rates with active rTMS compared with sham stimulation [19], suggesting that rTMS effectively alleviates depressive symptoms in BD. However, its efficacy in improving cognitive function remains controversial. While an rTMS study has demonstrated a mild-to-moderate cognitive improvement during the euthymic phase [20], this benefit has not been consistently observed during depressive episodes [21, 22].

Studies have demonstrated the antidepressant efficacy of H1-coil dTMS in patients with depression, including both unipolar and bipolar subtypes [23, 24, 25]. Its capability for deeper and broader cortical and subcortical stimulation allows it to modulate attention and cognitive-control networks, such as the fronto-parietal and fronto-limbic circuits [26, 27]. Such network modulation may facilitate cognitive enhancement beyond mood improvement alone. However, research on cognitive outcomes following dTMS treatment in BD remains limited. Current findings mainly originate from small-sample open-label studies or randomized controlled trials (RCTs) with inconsistent results [22, 25, 28].

Therefore, this study conducted a randomized, double-blind, and sham-controlled trial, using a comparatively large sample size, to systematically assess the efficacy of H1-coil dTMS on cognitive outcomes in BD patients. The objective is to provide robust clinical evidence supporting cognitive interventions in BD.

## 2. Materials and Methods

### 2.1 Study Design and Participants

This single-center, randomized, parallel-group, double-blind, sham-controlled clinical trial was implemented at Tianjin Anding Hospital, China (ClinicalTrials.gov identifier: NCT06524505), from August 2024 to September 2025. This manuscript focuses specifically on cognitive improvement and its relationship to changes in overall depressive symptoms. A more granular analysis of clinical symptoms (e.g., the 17-item Hamilton Depression Rating Scale (HDRS-17) subscales, anxiety measured by the Hamilton Anxiety Rating Scale) and biomarker data, as pre-registered, will be detailed in a forthcoming publication. The trial adhered to the Consolidated Standards of Reporting Trials (CONSORT) guidelines [29]. The trial consisted of two phases: (1) an acute treatment phase lasting 4 weeks, during which participants received 20 daily sessions of dTMS, excluding weekends; and (2) a follow-up phase lasting an additional 4 weeks without stimulation. Due to Coronavirus Disease 2019 (COVID-19)–related restrictions, conducting follow-up assessments after discharge proved challenging. Consequently, analyses were limited to data collected before and after the dTMS intervention.

### 2.2 Sample Size Calculation

A power analysis was carried out before enrollment to determine the required sample size, based on the medium effect size of dTMS on clinical efficacy in BD patients reported in the existing literature [30]. The analysis was calculated for the repeated-measures ANOVA inter-intra interaction. To detect between-group differences in changes of the HDRS-17 total score, a sample of 68 participants was estimated to provide 80% power (*p* < 0.05) to detect a medium-small effect size (Cohen’s *f* = 0.20, partial η^2^ = 0.04). The minimum required sample size was set at n = 80, allowing for a potential dropout rate of up to 15%, with participants allocated equally between groups. The sample size calculation was conducted using PASS software (version 14; NCSS, LLC, Kaysville, UT, USA).

### 2.3 Inclusion and Exclusion Criteria 

We enrolled inpatients from Department of Psychiatry at Tianjin Anding Hospital. All participants met the diagnostic criteria for bipolar I or II disorder during a current depressive phase. Antidepressant medication was prohibited during the study. Benzodiazepines were permitted, if necessary, up to 3 mg/day of lorazepam or an equivalent dose. Diagnoses were established by board-certified psychiatrists using the Diagnostic and Statistical Manual of Mental Disorders, Fifth Edition (DSM-5), and confirmed with the Mini-International Neuropsychiatric Interview (MINI).

The study included patients with the following characteristics: (1) aged 18 to 65 years; (2) a HDRS-17 total score of ≥17 [31]; (3) a stable medication regimen for 4 weeks or more before the treatment phase; and (4) for participants previously treated with antidepressants, a minimum 4-week washout period followed by re-evaluation. To enhance generalizability and increase the sample size, the study included patients meeting conventional criteria for TRBD (defined as non-response to at least two adequate medication trials of four weeks or longer) as well as those who remained symptomatic despite a stable monotherapy regimen of at least 4 weeks.

Exclusion criteria included: (1) a lifetime history of other psychiatric disorders, neurological diseases, or severe brain injury; (2) receipt of electroconvulsive therapy (ECT), rTMS, transcranial direct current stimulation (tDCS), transcranial alternating current stimulation, or other neurostimulation treatments within the past 3 months; (3) contraindications to magnetic stimulation (epilepsy, cardiovascular disorders, cranial metal implants); and (4) presence of hypomanic/manic symptoms or a Young Mania Rating Scale (YMRS) score higher than 12 at baseline. Requirements for participant withdrawal included: (1) missing two consecutive or a total of more than two stimulation sessions; (2) any change in medication regimen during the 4-week treatment phase; and (3) occurrence of severe adverse events throughout the acute stimulation phase.

### 2.4 Randomization, Concealment, and Blinding

**Fig. 1.**
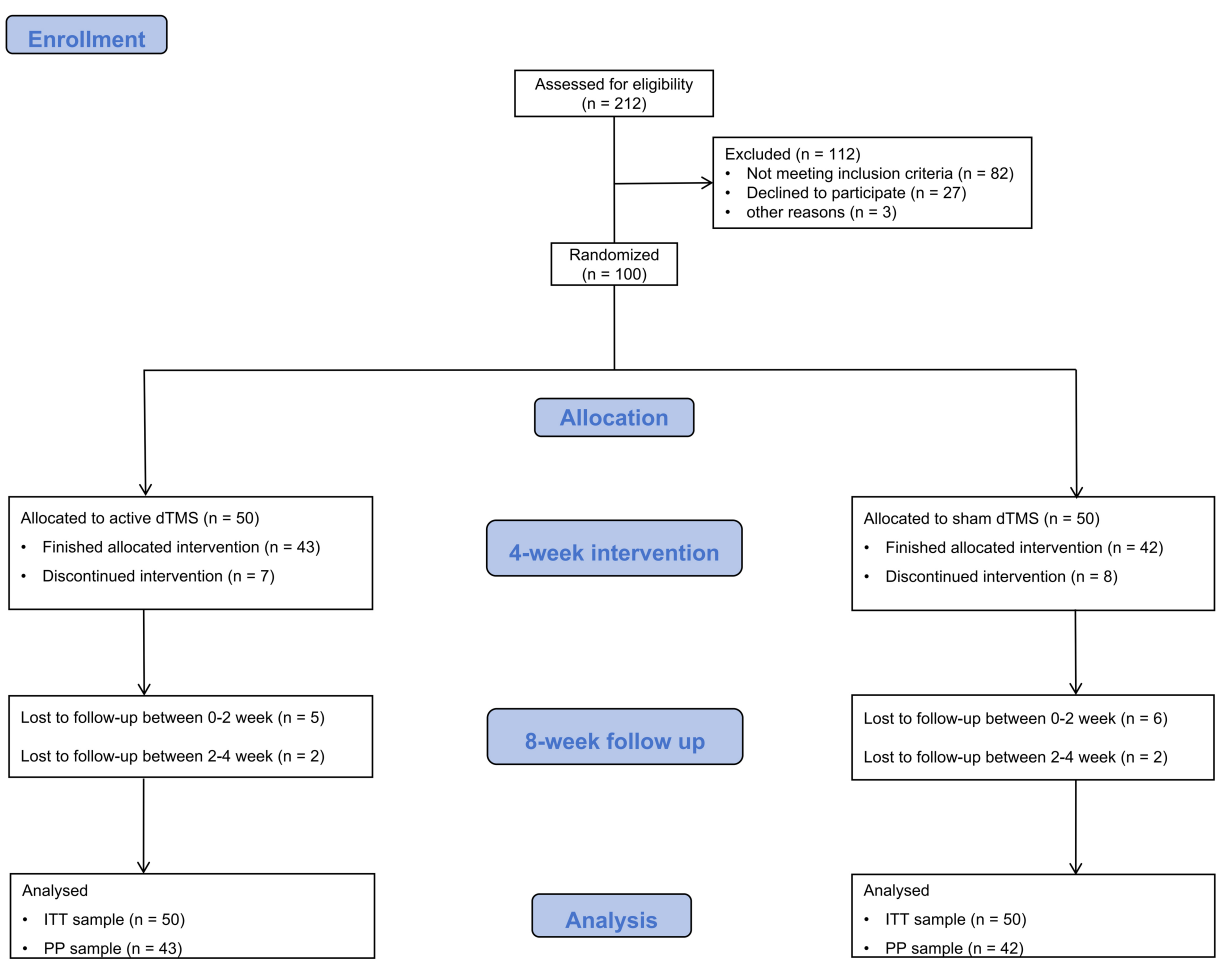
**CONSORT diagram showing the primary phases of the clinical trial**. dTMS, deep transcranial magnetic stimulation; ITT, intention-to-treat; PP, per-protocol; CONSORT, Consolidated Standards of Reporting Trials.

The CONSORT diagram for this study is presented in Fig. 1. Participants were randomly assigned in a 1:1 ratio to either active or sham dTMS. The allocation sequence was generated by an independent statistician via the PLAN protocol in SAS version 9.4 (SAS Institute Inc., Cary, NC, USA). A psychiatrist independent of the trial placed each assignment into sequentially numbered, sealed, opaque, and otherwise identical envelopes. At the point of randomization, the sealed envelope for each participant was opened by a study coordinator immediately before the first stimulation session to reveal the group allocation.

To maintain allocation concealment, the active and sham coils were coded as ‘Device A’ and ‘Device B’, respectively, throughout the trial. Only the device operators—who were not involved in participant assessment or data analysis—were aware of this code. Both devices were indistinguishable in appearance, sound, and scalp sensation, ensuring that participants and outcome raters remained blinded until the completion of all statistical analyses.

To assess blinding effectiveness, participants were asked after completing the treatment phase to guess their group assignment. The distribution of their responses between groups was compared with a chi-square test (or Fisher’s exact test, as appropriate) to assess whether their guesses deviated from chance.

### 2.5 Interventions

The intervention was administered using a deep transcranial magnetic stimulator (M-100 Ultimate, with an H1 coil; Shenzhen Yingchi Technology Co., Ltd., Shenzhen, Guangdong, China). The H1 coil was embedded in a helmet structure equipped with an active cooling system to maintain consistent performance throughout stimulation. A sham coil was designed to reproduce the tactile sensory experience of active stimulation while preventing neuronal stimulation. Before treatment initiation, each participant’s resting motor threshold (RMT) was determined, defined as the minimal stimulating intensity needed to yield a motor-evoked potential no less than 50 µV in 50% of attempts. The RMT was reassessed on the first treatment day and weekly thereafter. The stimulation coil was positioned 6 cm anterior to the individual’s motor “hot spot”, over the left DLPFC, identified using a ruler affixed to a fitted cap. Patients in the active dTMS group experienced a 4-week treatment course, consisting of 20 stimulation sessions administered once daily, 5 times a week. Each session comprised 55 trains of 18 Hz stimulation. Trains lasted 2 seconds each and were given at 120% of the resting motor threshold, with a 20-second pause between trains, resulting in 1980 pulses per session and 39,600 pulses per full treatment course. Participants in the sham group underwent identical procedures, except the stimulation was delivered through the sham coil, which did not produce cortical activation.

### 2.6 Assessments and Outcomes

Baseline demographic and clinical data included age, sex, years of education, marital status, occupational status, age at illness onset, current episode duration, diagnostic subtype, and current medication use. Cognitive assessments occurred at baseline and week 4, while clinical symptom evaluations took place at baseline, week 2, and week 4. The Measurement and Treatment Research to Improve Cognition in Schizophrenia (MATRICS) Consensus Cognitive Battery (MCCB) was applied to investigate cognitive function. Depressive and manic symptoms were evaluated using the HDRS-17 and YMRS, respectively. Adverse events were documented after each stimulation session.

The MCCB has been validated as a reliable instrument in the investigation of neurocognitive function in BD patients [32, 33]. The Chinese version used in this study has demonstrated good psychometric properties [34]. The MCCB comprises nine subtests that measure seven cognitive domains: (1) processing speed, assessed using the Brief Assessment of Cognition in Schizophrenia Symbol Coding (BACS-SC), Category Fluency (Animal Naming), and Trail Making Test–Part A (TMT-A); (2) attention/vigilance, measured by the Continuous Performance Test–Identical Pairs (CPT-IP); (3) working memory, evaluated with the Wechsler Memory Scale–III (WMS–III) Spatial Span; (4) verbal learning, tested via the Hopkins Verbal Learning Test–Revised (HVLT–R); (5) visual learning, assessed with the Brief Visuospatial Memory Test–Revised (BVMT–R); (6) reasoning and problem solving, measured by the Neuropsychological Assessment Battery (NAB) mazes; and (7) social cognition, evaluated using the Managing Emotions branch of the Mayer–Salovey–Caruso Emotional Intelligence Test (MSCEIT). Subtest scores were converted into standardized T-scores (mean = 50, SD = 10) using the MCCB computerized scoring program, adjusted for age, sex, and education. The global composite MCCB score is a standardized measure derived from the average of the seven domain scores.

The pre-specified primary outcome in the trial registry was the change from baseline to week 8 in the HDRS-17. Secondary outcomes included cognitive function, anxiety symptoms, and biomarker levels. For the present analysis, the change in MCCB scores from baseline to week 4 was considered the primary outcome. Secondary outcomes included: (1) change in HDRS-17 scores from baseline to week 4; (2) HDRS-17 response rate (≥50% decrease in scores from baseline) and HDRS-17 remission rate (scores ≤7) at weeks 2 and 4; and (3) incidence of adverse events. Other pre-registered secondary outcomes, including Hamilton Anxiety Rating Scale (HAMA) score, peripheral blood levels of brain-derived neurotrophic factor (BDNF) will be reported in a subsequent publication detailing the comprehensive clinical and biomarker profile of the trial. Four psychiatrists who received standardized training conducted all assessments. After training, intraclass correlation coefficients among raters for MCCB and HDRS-17 scores exceeded 0.80. Throughout the study, all raters remained blinded to group allocation.

### 2.7 Statistical Analysis

SPSS version 25.0 (SPSS Inc., Chicago, IL, USA) was used to analyze the data. All tests were two-tailed, with statistical significance set at *p* < 0.05. The normality of continuous variables was assessed using the Shapiro–Wilk test, and the homogeneity of variances was assessed using Levene’s test. To ensure group balance, differences in baseline demographic and clinical characteristics were evaluated. Differences in continuous variables were tested using independent-sample *t*-tests, and chi-squared or Fisher’s exact tests were used for categorical variables. The intention-to-treat (ITT) population included all randomized participants. We defined per-protocol (PP) completion as attendance at 80% or more of the treatment sessions. All data were analyzed on an ITT basis, and missing data were imputed via the last observation carried forward (LOCF) method. A two-way repeated-measures analysis of variance (RM-ANOVA) was conducted on cognitive outcomes. The model accounted for groups (active vs. sham) as the between-subjects parameter and time (baseline vs. week 4) as the within-subjects parameter. The MCCB T-scores, which are normed for age, sex, and education, were used as the outcome measures. The RM-ANOVA was adjusted only for baseline HDRS scores to control for initial depression severity. Additionally, a PP analysis was performed to assess the robustness of the results.

To explore the impact of approaches to handling missing MCCB data, sensitivity analyses were conducted, using multiple imputation and a worst-case scenario analysis. For the multiple imputation, 20 imputed datasets were created using the Fully Conditional Specification (FCS) method in R (“mice” package). A repeated-measures ANOVA model was fitted to each imputed dataset, and the results were pooled using Rubin’s rules to generate the final estimates. For the worst-case scenario analysis, missing data were imputed to represent clinical deterioration, defined a priori as a decline of ≥0.5 standard deviations from the participant’s own baseline score. To control for potential inflation of Type I error due to multiple comparisons across MCCB subtests, a false discovery rate (FDR) correction was applied. Changes in HDRS-17 scores within the ITT sample were analyzed using the identical statistical approach applied to the primary outcomes, across baseline, week 2, and week 4. The sphericity assumption for the within-subject effects was evaluated using Mauchly’s test. In case of violation (*p* < 0.05), the Greenhouse-Geisser correction was applied. In the instance of robust significance group and time interaction, post-hoc tests with Bonferroni corrections were conducted. In the absence of significance, no further tests were carried out. The effect size for the RM-ANOVA tests was estimated using partial eta-squared (η_p_^2^). Response and remission rates were compared using χ^2^ tests. To explore the association between improvements in depression (baseline minus endpoint scores) and cognition (endpoint minus baseline scores), Pearson’s correlation analysis was performed.

## 3. Results

### 3.1 Demographic Information

As shown in Fig. 1, 212 participants were screened, of whom 112 were excluded for various reasons. Finally, 100 participants were enrolled and randomized. A total of 85 participants completed the 4-week intervention. In the active group, five participants withdrew between baseline and week 2 (two due to missing two treatment sessions, two due to adverse events such as headache, and one withdrew consent), and two more withdrew between weeks 2 and 4 (due to perceived lack of improvement). In the sham group, six participants withdrew between baseline and week 2 (two due to missing two treatment sessions, three due to perceived lack of improvement, and one due to withdrawn consent). Two additional participants withdrew between weeks 2 and 4 (due to perceived lack of improvement). The dropout rates did not differ significantly between groups (*p* = 0.78).

Of the enrolled participants, 68 were female (68%), and 32 were male (32%), with a mean age of 38.65 years (SD = 15.91). There were no significant differences in baseline demographic or clinical characteristics between groups (Table 1). Additionally, baseline characteristics were comparable between completers and non-completers (**Supplementary Table 1**).

**Table 1.** **Baseline demographic and clinical characteristics of the sample**.

		Active dTMS (n = 50)	Sham dTMS (n = 50)	*t/$\chi^{2}$*	*p*
Age, mean ± SD, years		36.84 ± 14.62	41.86 ± 15.18	–1.68	0.10
Sex, n (% Female)		30 (60)	38 (76)	2.94	0.09
Marital status, n (% Married)		28 (56)	31 (62)	0.37	0.54
Occupational status, n (% Employed)		18 (36)	21 (42)	0.38	0.54
Educational level, mean ± SD, years		12.78 ± 3.76	12.83 ± 3.04	–0.07	0.94
Age at illness onset, mean ± SD, years		21.04 ± 6.77	23.38 ± 8.33	–1.54	0.13
Current episode duration, mean ± SD, months		4.23 ± 4.33	5.25 ± 6.25	–0.95	0.35
Diagnosis subtype, n (%)					
	Bipolar Disorder Type I	22 (44)	24 (48)	0.16	0.69
	Bipolar Disorder Type II	28 (56)	26 (52)	0.16	0.69
Current medication use, n (%)					
	First-line therapy	46 (92)	43 (86)	0.92	0.34
	Lithium	29 (58)	32 (64)	0.38	0.54
	Valproate	9 (18)	10 (20)	0.07	0.80
	Lamotrigine	15 (30)	17 (34)	0.18	0.67
	Quetiapine	24 (48)	16 (32)	2.67	0.10
	Benzodiazepine	23 (46)	16 (32)	2.06	0.15
HDRS-17 score, mean ± SD		24.90 ± 7.14	23.76 ± 5.07	0.92	0.36
YMRS score, mean ± SD		3.48 ± 5.55	3.04 ± 4.51	0.44	0.66

HDRS-17, 17-item Hamilton Depression Rating Scale; YMRS, Young Mania Rating Scale.

### 3.2 Cognitive Function

**Fig. 2.**
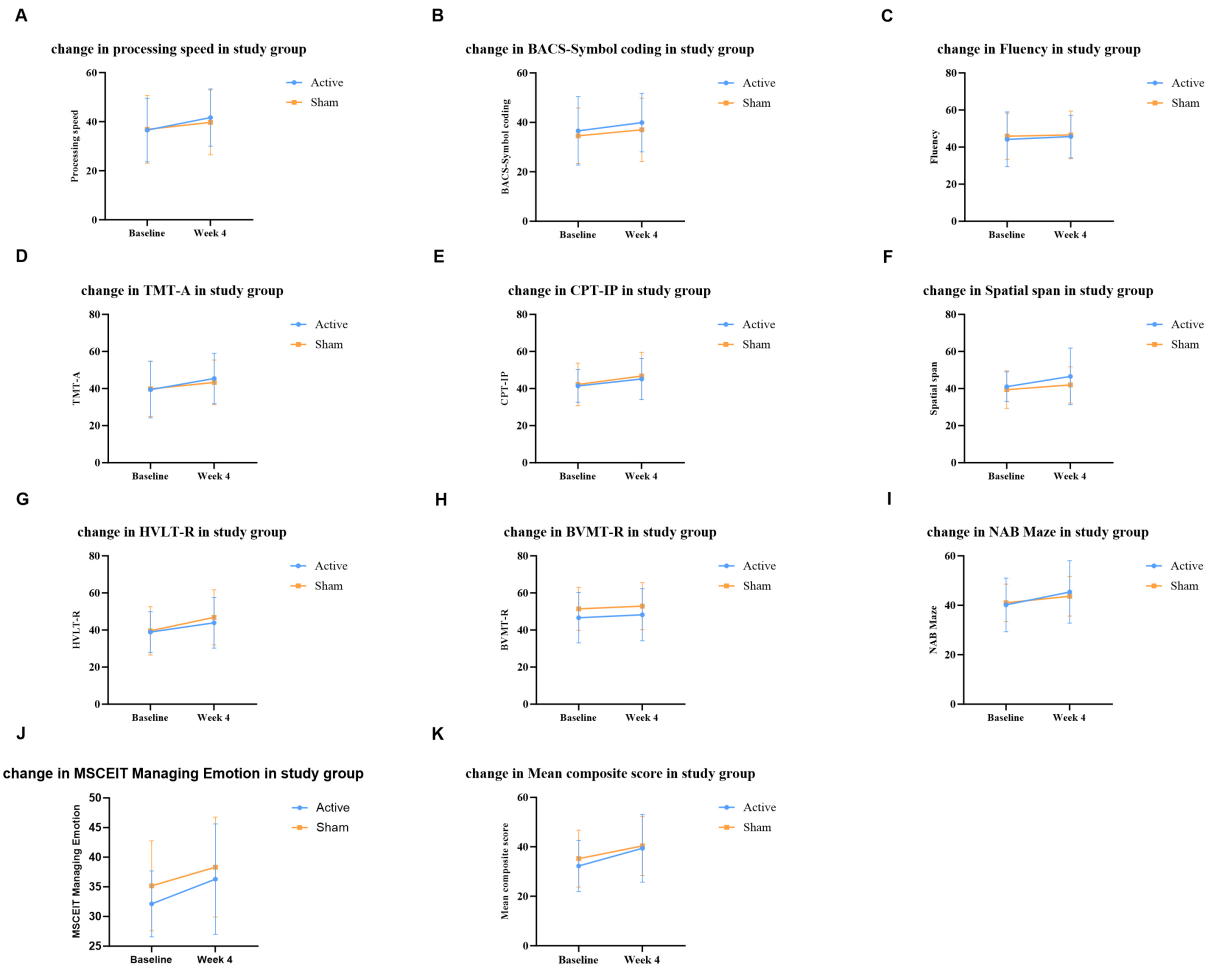
**Comparison of cognitive scores between groups at baseline and week 4**. (A) Processing speed changes over time. (B) BACS-Symbol Coding changes over time. (C) Fluency changes over time. (D) TMT-A changes over time. (E) CPT-IP changes over time. (F) Spatial Span changes over time. (G) HVLT–R changes over time. (H) BVMT–R changes over time. (I) NAB mazes change over time. (J) MSCEIT Managing Emotion changes over time. (K) Mean composite score changes over time. BACS, Brief Assessment of Cognition in Schizophrenia; TMT-A, Trail Making Test–Part A; CPT-IP, Continuous Performance Test–Identical Pairs; HVLT-R, Hopkins Verbal Learning Test–Revised; BVMT-R, Brief Visuospatial Memory Test–Revised; NAB-mazes, Neuropsychological Assessment Battery–mazes; MSCEIT, Mayer–Salovey–Caruso Emotional Intelligence Test.

After the 4-week intervention, all cognitive domains except visual learning showed significant changes (*p* < 0.05; Fig. 2 and **Supplementary Table 2**). A RM-ANOVA revealed a significant main effect of time on the mean composite score (*F*(1, 97) = 49.18, *p* < 0.001, η_p_^2^ = 0.336, 95% CI [0.189, 0.460]). Significant time effects were also observed in processing speed (*F*(1, 97) = 22.72, *p* < 0.001, η_p_^2^ = 0.190, 95% CI [0.068, 0.320]), attention/vigilance (*F*(1, 97) = 25.68, *p* < 0.001, η_p_^2^ = 0.209, 95% CI [0.081, 0.340]), working memory (*F*(1, 97) = 9.00, *p* = 0.003, η_p_^2^ = 0.085, 95% CI [0.010, 0.201]), verbal learning (*F*(1, 97) = 28.46, *p* < 0.001, η_p_^2^ = 0.227, 95% CI [0.095, 0.357]), reasoning and problem solving (*F*(1, 97) = 18.32, *p* < 0.001, η_p_^2^ = 0.159, 95% CI [0.047, 0.287]), and social cognition (*F*(1, 97) = 21.73, *p* < 0.001, η_p_^2^ = 0.183, 95% CI [0.063, 0.313]). In contrast, visual learning showed no significant change over time (*F*(1, 97) = 2.75, *p* = 0.101, η_p_^2^ = 0.028, 95% CI [0.000, 0.117]). All significant findings remained significant after FDR correction (*p* < 0.05). From baseline to endpoint, notable improvements were observed in both groups across most cognitive domains (*p* < 0.05). However, no significant main effect of group and no significant time × group interaction were observed (all *p* > 0.05).

A per-protocol analysis confirmed the robustness of the primary results. Repeated measures ANOVA revealed significant main effects of time on processing speed, attention/vigilance, working memory, verbal learning, reasoning, social cognition, and the mean composite score (all *p* < 0.05; **Supplementary Table 3**). However, neither a significant main effect of group nor a significant time × group interaction was observed (all *p* > 0.05; **Supplementary Table 3**), indicating that the extent of cognitive improvement was similar between the active and sham groups. These findings were consistent in both the multiple imputation analysis (**Supplementary Table 4**) and the worst-case scenario analysis (**Supplementary Table 5**).

No significant correlations were observed between changes in HDRS-17 scores and changes in MCCB composite or domain scores: mean composite score (*r* = 0.24, *p* = 0.10), information processing speed (*r* = 0.02, *p* = 0.90), attention/vigilance (*r* = 0.11, *p* = 0.44), working memory (*r* = 0.01, *p* = 0.97), verbal learning (*r* = 0.22, *p* = 0.13), visual learning (*r* = 0.22, *p* = 0.14), reasoning and problem solving (*r* = 0.22, *p* = 0.13), or social cognition (*r* = 0.16, *p* = 0.28).

### 3.3 Depressive Symptoms

Analysis of HDRS-17 total scores revealed a significant group × time interaction (*F*(1.86, 182.05) = 7.80, *p* = 0.001, η_p_^2^ = 0.074, 95% CI [0.015, 0.146]) and a robust main time effect (*F*(1.86, 182.05) = 173.50, *p* < 0.001, η_p_^2^ = 0.639, 95% CI [0.559, 0.695]). The degrees of freedom for these effects are reported using the Greenhouse-Geisser correction because Mauchly’s test indicated a violation of the sphericity assumption (W = 0.92, $\chi^{2}$(2) = 7.73, *p* = 0.02). No significant main effect of group was found (*F*(1, 98) = 1.46, *p* = 0.23, η_p_^2^ = 0.015, 95% CI [0.000, 0.091]). Post hoc tests showed that HDRS-17 scores were significantly lower in the active dTMS group compared to the sham group at week 4 (mean difference = 2.94, 95% CI [0.10, 5.78], *p* = 0.04), but not at week 2 (*p* > 0.05; Table 2).

**Table 2.** **Comparison of HDRS-17 scores between groups at baseline, week 2, and week 4**.

Group	HDRS-17 score, mean ± SD			*F*_Group_ (*p*_Group_)	*F*_time_ (*p*_time_)	*F*_Group*time_ (*p*_Group*time_)
	Baseline	Week 2	Week 4			
Active dTMS	24.90 ± 7.14	15.80 ± 8.51	12.50 ± 7.86	1.46 (0.23)	173.50 (<**0.001**)	7.80 (**0.001**)
Sham dTMS	23.76 ± 5.07	18.36 ± 5.53	15.44 ± 6.36			

Bold values indicate *p* < 0.05.

At week 4, the active group showed a significant superiority over the sham group in terms of response rate (50% vs. 24%; OR = 3.17, 95% CI [1.35, 7.44], *p* = 0.007), although the difference was not significant at week 2 (28% vs. 14%; OR = 2.39, 95% CI [0.87, 6.56], *p* = 0.09). No significant differences in remission rates were observed between groups at week 2 or week 4 (Table 3).

**Table 3.** **Comparison of response and remission rates between groups**.

		n (%)	OR [95% CI]	*p*
Response (active × sham)				
	Week 2	14 (28.0) vs. 7 (14.0)	2.39 (0.87–6.56)	0.090
	Week 4	25 (50.0) vs. 12 (24.0)	3.17 (1.35–7.44)	**0.007**
Remission (active × sham)				
	Week 2	10 (20.0) vs. 7 (14.0)	1.54 (0.53–4.42)	0.420
	Week 4	15 (30.0) vs. 10 (20.0)	1.71 (0.68–4.30)	0.250

Bold values indicate *p* < 0.05.

### 3.4 Safety Measures

No severe adverse events (AEs) were reported during the trial. The overall incidence of AEs did not differ significantly between the active dTMS and sham groups (*p* > 0.05). Adverse events in the active dTMS group included headache (n = 3), insomnia (n = 2), local discomfort (n = 2), and muscle twitching (n = 1). In the sham group, the reported events included insomnia (n = 3), headache (n = 2), and local discomfort (n = 1), with no muscle twitching. Most mild AEs resolved within 2 hours after the intervention, and those with more pronounced symptoms resolved spontaneously within 2 days without additional treatment. Vital signs remained stable, and no participants experienced seizures or manic symptoms.

### 3.5 Integrity of Blinding

At study completion, blinding integrity was evaluated for participants and assessors. Among participants, 25 of 43 (58.14%) in the active group and 22 of 42 (52.38%) in the sham group believed they had received active dTMS. Chi-square analysis showed no significant difference in correct guesses between groups (χ^2^ = 0.98, *p* = 0.32). Among evaluators, 26 patients of the active group (26/43) and 22 in the sham group (22/42) were correctly identified. Similarly, no statistical difference was detected in the accuracy of blinding between groups (χ^2^ = 0.56, *p* = 0.45).

## 4. Discussion

This study, to our knowledge, represents the most extensive randomized, double-blind, sham-controlled trial systematically evaluating the effects of H1-coil dTMS on cognitive function and clinical symptoms in BD patients. We found no evidence of specific cognitive enhancement beyond sham stimulation; however, substantial and comparable cognitive gains were observed in both groups post-intervention. Regarding depressive symptoms, the data suggest a modest benefit of active dTMS: the between-group difference at week 4 was 2.94 points on the HDRS-17, approaching the conventional minimal clinically important difference (MCID) of 3 points. The response rate was also numerically higher in the active group (50% vs. 24%). The intervention demonstrated a favorable safety profile.

### 4.1 Changes in Cognitive Function

This study has demonstrated that active dTMS improved multiple cognitive domains in patients with BD, consistent with previous findings [28]. An Earlier open-label study reported that dTMS enhanced reaction time and spatial working memory, approaching levels of healthy controls [22]. A critical question is whether the broad-based cognitive improvements resulted directly from dTMS or were mediated by mood improvement. Given the lack of statistical significance between the study groups, a more plausible explanation involves nonspecific factors, such as practice effects from repeated neuropsychological testing [35] and the clinical benefits of trial participation itself, including structured care and positive expectancies. The absence of a significant correlation between changes in depression scores and cognition changes, consistent with a prior study [28], further supports this interpretation. These findings indicate that the trait-like cognitive deficits in BD may be relatively resistant to change with the current dTMS protocol.

In this study, no significant improvement occurred in visual learning, consistent with findings in MDD [24]. Notably, visual learning scores in patients with BD were not low at baseline, approaching levels of healthy controls after treatment. Hence, a significant improvement after treatment was not expected.

The cognitive effects of dTMS in depressive patients remain unclear. While some small open-label studies reported improvements in memory, attention, and processing speed [36, 37], the cognitive gains observed after 20 dTMS sessions in our trial—similar to previous studies in both unipolar and bipolar depression [25]—were not specific to the active group. Comparable results have also been reported in schizophrenia [38] and MDD [24]. Several factors should be considered. First, the H1 coil produces a relatively diffuse stimulation pattern. It primarily targets the left DLPFC but also delivers weaker stimulation to the contralateral DLPFC and other prefrontal regions, potentially limiting improvement in specific cognitive domains [39]. Second, high-frequency stimulation of the left DLPFC is well-known for its antidepressant efficacy through modulation of mood-regulatory circuits, such as the DLPFC–anterior cingulate pathway [40]. In contrast, higher-order cognitive control, which involves distinct DLPFC-parietal connections, may require different stimulation parameters for optimal effect [41]. Neuroimaging studies suggest that rTMS/dTMS over the DLPFC mainly influences default-mode circuits implicated in emotional regulation, with less consistent effects on the cognitive-control networks [42]. Therefore, stimulation parameters optimized for mood improvement may be suboptimal for engaging the networks underlying cognitive function. While one line of research has predominantly focused on the DLPFC, regions like the orbitofrontal cortex (OFC) and the dorsomedial prefrontal cortex (DMPFC) also play critical roles in cognitive processes [43]. Identifying optimal brain regions for cognitive enhancement is essential. Third, stimulation frequency and duration may have been insufficient to elicit significant cognitive effects. Previous research suggests accelerated or prolonged interventions (e.g., twice-daily dTMS) yield mild to moderate improvements across cognitive measures, with differential outcomes depending on treatment duration [44]. Finally, the MCCB was initially developed for schizophrenia. Although widely used in BD research, evidence indicates that it might lack sensitivity for detecting subtle cognitive changes in BD patients, particularly those who are euthymic or have residual depressive symptoms [45, 46]. For example, smaller effect sizes have been shown in distinguishing individuals with BD from healthy controls using the MCCB compared to its use in schizophrenia [47]. Thus, genuine cognitive effects in our study may have been obscured by the tool’s limited sensitivity. Future studies should include cognitive assessments more specific and sensitive to affective disorders, such as tasks involving emotional stimuli.

A network meta-analysis demonstrated that left DLPFC rTMS greatly improves global cognition in individuals with mild cognitive impairment (standardized mean difference [SMD] = 1.25, 95% CI [0.57, 1.93]). This suggests that future dTMS studies might benefit from focusing on individuals with more pronounced cognitive deficits, thereby mitigating potential ceiling effects. Furthermore, preconditioning with tDCS enhances cortical plasticity induced by rTMS [48]. Thus, combination therapies might improve cognition. A recent multimodal neuromodulation study highlighted the potential benefits of combining TMS with tDCS in improving cognitive function and depressive symptoms among BD patients [49]. These findings imply that stimulation depth and spatial reach may differentially modulate cortical and subcortical circuits involved in mood and cognition. Compared to superficial stimulation methods, dTMS primarily targets deeper fronto-limbic networks, which may help explain its more pronounced antidepressant effects relative to its cognitive benefits in this study [26].

The cognitive-enhancing effects of rTMS may result from direct modulation of regional brain activity or optimization of neural network connectivity. For instance, rTMS has been shown to promote dopamine release in the striatum and caudate nucleus and to strengthen functional connectivity between the DLPFC and key subcortical regions, such as the anterior cingulate cortex and striatum. These neurophysiological changes may collectively enhance cognitive processing speed, increase neural efficiency, and promote cortical plasticity, thereby accelerating cognitive operations [50, 51, 52]. However, the exact mechanisms underlying dTMS**-**induced cognitive improvements require further investigation through neuroimaging, genetic, and biochemical studies.

### 4.2 Changes in Depressive Symptoms

Consistent with previous research, this trial provides further evidence supporting the potential efficacy and acceptability of dTMS for treating depressive symptoms in BD. Our findings indicate that dTMS was associated with therapeutic benefit and was well-tolerated. After the 4-week intervention, a robust response rate was observed in the active dTMS group (50%), which was significantly higher than in the sham group (24%), aligning closely with previous reports for rTMS in BD (44.3% response rate) [53]. In addition, high-frequency dTMS combined with pharmacotherapy in depressed patients has shown a favorable response (70.73%) and remission rates (19.51%), without significant variation across depression subtypes [44]. While the mean endpoint score difference was modest and sub-MCID, the significant group × time interaction and the meaningful week-4 separation in responder rates together suggest a specific therapeutic effect of active dTMS, albeit of limited magnitude. The observed clinical improvement likely represents the combined contribution of this specific effect and nonspecific factors. Regarding sustainability, the long-term efficacy of dTMS could not be assessed in this study due to the lack of follow-up data. However, existing research suggests that therapeutic effects diminish within weeks if no maintenance dTMS sessions are provided [28]. Conversely, maintenance dTMS has sustained improvements in HDRS scores over 6–12 months [54]. Thus, incorporating maintenance treatment in clinical practice may be essential to consolidate therapeutic gains.

### 4.3 Limitations

Several limitations of this study should be acknowledged. First, outcomes were assessed only during the acute treatment period, and the COVID-19 pandemic prevented post-treatment follow-up assessments, limiting our evaluation of the long-term sustainability of therapeutic effects. Second, the statistically significant between-group difference in HDRS-17 scores was modest in magnitude (approximately 3 points), and its clinical meaningfulness requires further confirmation in larger trials. Third, the study relied on behavioral and clinical measures without incorporating neurophysiological (e.g., Transcranial Magnetic Stimulation-Electroencephalography (EEG)) or neuroimaging (e.g., functional Magnetic Resonance Imaging (fMRI) biomarkers. Consequently, we could not objectively verify the targeted cortical engagement or directly investigate the neural mechanisms underlying the clinical effects. Fourth, this was a single-center trial undertaken primarily in an inpatient setting, which may limit generalizability. Potential confounding from concomitant medication at baseline also cannot be fully excluded. Fifth, the lack of a healthy comparator precludes the determination of baseline cognitive impairment and whether cognitive function fully recovered after intervention. In addition, the trial was powered for clinical rather than cognitive endpoints, and subtle cognitive effects may therefore have been underdetected. Finally, an important methodological consideration concerns the reporting strategy. To enable a focused and in-depth investigation of cognitive effects—a core aim of this study—we centered the analysis on the MCCB. Consequently, the pre-registered primary outcome (HDRS-17) was analyzed and is reported in this manuscript as a secondary outcome, specifically to examine its relationship with cognitive change. Data for other protocol-specified outcomes (HAMA, BDNF) are reserved for a future paper on overall clinical and biomarker profiles. This sequential and reprioritized reporting approach represents a deviation from the initial protocol. This approach was adopted to enhance the clarity and thematic coherence of the present cognitive-focused manuscript. All pre-registered data will be fully disclosed across the planned series of publications. Future studies should incorporate long-term follow-up, healthy control comparisons, and multimodal biomarkers to clarify the independent and sustained effects of dTMS on both cognition and mood symptoms.

## 5. Conclusions

In conclusion, this large RCT indicates that H1‑coil dTMS is a well-tolerated and cognitively safe intervention for BD. Critically, it produced no specific cognitive enhancement, suggesting that the observed improvements may involve nonspecific effects. Regarding depressive symptoms, active dTMS was associated with modest but statistically significant clinical benefits compared to sham, reflected in lower depressive symptom scores and a higher response rate at the study endpoint. Together, these findings support the potential of dTMS as a safe adjunctive treatment for BD, offering a measurable, albeit limited, benefit without adding cognitive burden; thus, it presents a viable option, particularly when pharmacotherapy is limited or poorly tolerated. To enhance translational applications, future research should extend follow-up periods and systematically investigate stimulation parameters. Furthermore, the integration of multimodal assessments (e.g., fMRI, EEG) with cognitive remediation may help identify predictors of treatment response and refine personalized protocols, thereby potentially improving therapeutic outcomes for patients.

## Availability of Data and Materials

The data that support the findings of this study are available from the corresponding author upon reasonable request.

## Supplementary Material

Supplementary material associated with this article can be found, in the online version, at https://doi.org/10.31083/AP47409.

## References

[1] Ferrari AJ, Stockings E, Khoo JP, Erskine HE, Degenhardt L, Vos T (2016). The prevalence and burden of bipolar disorder: findings from the Global Burden of Disease Study 2013. *Bipolar Disorders*.

[2] Belmaker RH, Bersudsky Y (2004). Bipolar disorder: Mania and depression. *Discovery Medicine*.

[3] Judd LL, Akiskal HS, Schettler PJ, Endicott J, Maser J, Solomon DA (2002). The long-term natural history of the weekly symptomatic status of bipolar I disorder. *Archives of General Psychiatry*.

[4] Rosa AR, Reinares M, Michalak EE, Bonnin CM, Sole B, Franco C (2010). Functional impairment and disability across mood states in bipolar disorder. *Value in Health: the Journal of the International Society for Pharmacoeconomics and Outcomes Research*.

[5] Volkert J, Schiele MA, Kazmaier J, Glaser F, Zierhut KC, Kopf J (2016). Cognitive deficits in bipolar disorder: from acute episode to remission. *European Archives of Psychiatry and Clinical Neuroscience*.

[6] Cipriani G, Danti S, Carlesi C, Cammisuli DM, Di Fiorino M (2017). Bipolar Disorder and Cognitive Dysfunction: A Complex Link. *The Journal of Nervous and Mental Disease*.

[7] Demmo C, Lagerberg TV, Kvitland LR, Aminoff SR, Hellvin T, Simonsen C (2018). Neurocognitive functioning, clinical course and functional outcome in first-treatment bipolar I disorder patients with and without clinical relapse: A 1-year follow-up study. *Bipolar Disorders*.

[8] Tian L, Liu Y, Xu J, Mao Z, Xing X, Bo Q (2025). Neurocognitive function across different phases of bipolar disorder: an evaluation using the B-CATS. *Frontiers in Psychiatry*.

[9] Bo Q, Mao Z, Li X, Wang Z, Wang C, Ma X (2017). Use of the MATRICS consensus cognitive battery (MCCB) to evaluate cognitive deficits in bipolar disorder: A systematic review and meta-analysis. *PloS One*.

[10] Ehrminger M, Brunet-Gouet E, Cannavo AS, Aouizerate B, Cussac I, Azorin JM (2021). Longitudinal relationships between cognition and functioning over 2 years in euthymic patients with bipolar disorder: a cross-lagged panel model approach with the FACE-BD cohort. *The British Journal of Psychiatry: the Journal of Mental Science*.

[11] Schouws SNTM, Comijs HC, Dols A, Beekman ATF, Stek ML (2016). Five-year follow-up of cognitive impairment in older adults with bipolar disorder. *Bipolar Disorders*.

[12] Ishisaka N, Shimano S, Miura T, Motomura K, Horii M, Imanaga H (2017). Neurocognitive profile of euthymic Japanese patients with bipolar disorder. *Psychiatry and Clinical Neurosciences*.

[13] Budde M, Schulze TG (2014). Neurocognitive correlates of the course of bipolar disorder. *Harvard Review of Psychiatry*.

[14] Sparding T, Joas E, Clements C, Sellgren CM, Pålsson E, Landén M (2021). Long-term trajectory of cognitive performance in people with bipolar disorder and controls: 6-year longitudinal study. *BJPsych Open*.

[15] MacQueen GM, Memedovich KA (2017). Cognitive dysfunction in major depression and bipolar disorder: Assessment and treatment options. *Psychiatry and Clinical Neurosciences*.

[16] Wingo AP, Wingo TS, Harvey PD, Baldessarini RJ (2009). Effects of lithium on cognitive performance: a meta-analysis. *The Journal of Clinical Psychiatry*.

[17] Gao K (2024). Role of Electroconvulsive Therapy, Ketamine Infusion, and Deep Repetitive Transcranial Magnetic Stimulation in Treatment-Resistant Bipolar Depression: A Case Report. *Medicina (Kaunas, Lithuania)*.

[18] Pandya M, Altinay M, Malone DA, Anand A (2012). Where in the brain is depression?. *Current Psychiatry Reports*.

[19] Nguyen TD, Hieronymus F, Lorentzen R, McGirr A, Østergaard SD (2021). The efficacy of repetitive transcranial magnetic stimulation (rTMS) for bipolar depression: A systematic review and meta-analysis. *Journal of Affective Disorders*.

[20] Yang LL, Zhao D, Kong LL, Sun YQ, Wang ZY, Gao YY (2019). High-frequency repetitive transcranial magnetic stimulation (rTMS) improves neurocognitive function in bipolar disorder. *Journal of Affective Disorder*.

[21] Hu SH, Lai JB, Xu DR, Qi HL, Peterson BS, Bao AM (2016). Efficacy of repetitive transcranial magnetic stimulation with quetiapine in treating bipolar II depression: a randomized, double-blinded, control study. *Scientific Reports*.

[22] Harel EV, Zangen A, Roth Y, Reti I, Braw Y, Levkovitz Y (2011). H-coil repetitive transcranial magnetic stimulation for the treatment of bipolar depression: an add-on, safety and feasibility study. *The World Journal of Biological Psychiatry: the Official Journal of the World Federation of Societies of Biological Psychiatry*.

[23] Levkovitz Y, Isserles M, Padberg F, Lisanby SH, Bystritsky A, Xia G (2015). Efficacy and safety of deep transcranial magnetic stimulation for major depression: a prospective multicenter randomized controlled trial. *World Psychiatry: Official Journal of the World Psychiatric Association (WPA)*.

[24] Kaster TS, Daskalakis ZJ, Noda Y, Knyahnytska Y, Downar J, Rajji TK (2018). Efficacy, tolerability, and cognitive effects of deep transcranial magnetic stimulation for late-life depression: a prospective randomized controlled trial. *Neuropsychopharmacology: Official Publication of the American College of Neuropsychopharmacology*.

[25] Matsuda Y, Kito S, Igarashi Y, Shigeta M (2020). Efficacy and Safety of Deep Transcranial Magnetic Stimulation in Office Workers with Treatment-Resistant Depression: A Randomized, Double-Blind, Sham-Controlled Trial. *Neuropsychobiology*.

[26] Cheng JL, Tan C, Liu HY, Han DM, Liu ZC (2023). Past, present, and future of deep transcranial magnetic stimulation: A review in psychiatric and neurological disorders. *World Journal of Psychiatry*.

[27] Martin DM, Su Y, Chan HF, Dielenberg V, Chow E, Xu M (2024). Individualised Transcranial Magnetic Stimulation Targeting of the Left Dorsolateral Prefrontal Cortex for Enhancing Cognition: A Randomised Controlled Trial. *Brain Sciences*.

[28] Myczkowski ML, Fernandes A, Moreno M, Valiengo L, Lafer B, Moreno RA (2018). Cognitive outcomes of TMS treatment in bipolar depression: Safety data from a randomized controlled trial. *Journal of Affective Disorders*.

[29] Stevely A, Dimairo M, Todd S, Julious SA, Nicholl J, Hind D (2015). An Investigation of the Shortcomings of the CONSORT 2010 Statement for the Reporting of Group Sequential Randomised Controlled Trials: A Methodological Systematic Review. *PloS One*.

[30] Tavares DF, Myczkowski ML, Alberto RL, Valiengo L, Rios RM, Gordon P (2017). Treatment of Bipolar Depression with Deep TMS: Results from a Double-Blind, Randomized, Parallel Group, Sham-Controlled Clinical Trial. *Neuropsychopharmacology: Official Publication of the American College of Neuropsychopharmacology*.

[31] HAMILTON M (1960). A rating scale for depression. *Journal of Neurology, Neurosurgery, and Psychiatry*.

[32] Burdick KE, Russo M, Frangou S, Mahon K, Braga RJ, Shanahan M (2014). Empirical evidence for discrete neurocognitive subgroups in bipolar disorder: clinical implications. *Psychological Medicine*.

[33] Sperry SH, O’Connor LK, Öngür D, Cohen BM, Keshavan MS, Lewandowski KE (2015). Measuring Cognition in Bipolar Disorder with Psychosis Using the MATRICS Consensus Cognitive Battery. *Journal of the International Neuropsychological Society: JINS*.

[34] Shi C, Kang L, Yao S, Ma Y, Li T, Liang Y (2015). The MATRICS Consensus Cognitive Battery (MCCB): Co-norming and standardization in China. *Schizophrenia Research*.

[35] Burdick KE, Ketter TA, Goldberg JF, Calabrese JR (2015). Assessing cognitive function in bipolar disorder: challenges and recommendations for clinical trial design. *The Journal of Clinical Psychiatry*.

[36] Bersani FS, Minichino A, Enticott PG, Mazzarini L, Khan N, Antonacci G (2013). Deep transcranial magnetic stimulation as a treatment for psychiatric disorders: a comprehensive review. *European Psychiatry: the Journal of the Association of European Psychiatrists*.

[37] Kedzior KK, Gierke L, Gellersen HM, Berlim MT (2016). Cognitive functioning and deep transcranial magnetic stimulation (DTMS) in major psychiatric disorders: A systematic review. *Journal of Psychiatric Research*.

[38] Rabany L, Deutsch L, Levkovitz Y (2014). Double-blind, randomized sham controlled study of deep-TMS add-on treatment for negative symptoms and cognitive deficits in schizophrenia. *Journal of Psychopharmacology (Oxford, England)*.

[39] Roth Y, Amir A, Levkovitz Y, Zangen A (2007). Three-dimensional distribution of the electric field induced in the brain by transcranial magnetic stimulation using figure-8 and deep H-coils. *Journal of Clinical Neurophysiology: Official Publication of the American Electroencephalographic Society*.

[40] Cole EJ, Phillips AL, Bentzley BS, Stimpson KH, Nejad R, Barmak F (2022). Stanford Neuromodulation Therapy (SNT): A Double-Blind Randomized Controlled Trial. *The American Journal of Psychiatry*.

[41] Niendam TA, Laird AR, Ray KL, Dean YM, Glahn DC, Carter CS (2012). Meta-analytic evidence for a superordinate cognitive control network subserving diverse executive functions. *Cognitive, Affective & Behavioral Neuroscience*.

[42] Liston C, Chen AC, Zebley BD, Drysdale AT, Gordon R, Leuchter B (2014). Default mode network mechanisms of transcranial magnetic stimulation in depression. *Biological Psychiatry*.

[43] Phillips ML, Swartz HA (2014). A critical appraisal of neuroimaging studies of bipolar disorder: toward a new conceptualization of underlying neural circuitry and a road map for future research. *The American Journal of Psychiatry*.

[44] Rapinesi C, Kotzalidis GD, Ferracuti S, Girardi N, Zangen A, Sani G (2018). Add-on high frequency deep transcranial magnetic stimulation (dTMS) to bilateral prefrontal cortex in depressive episodes of patients with major depressive disorder, bipolar disorder I, and major depressive with alcohol use disorders. *Neuroscience Letters*.

[45] Miskowiak KW, Burdick KE, Martinez-Aran A, Bonnin CM, Bowie CR, Carvalho AF (2017). Methodological recommendations for cognition trials in bipolar disorder by the International Society for Bipolar Disorders Targeting Cognition Task Force. *Bipolar Disorders*.

[46] Zhu Y, Womer FY, Leng H, Chang M, Yin Z, Wei Y (2019). The Relationship Between Cognitive Dysfunction and Symptom Dimensions Across Schizophrenia, Bipolar Disorder, and Major Depressive Disorder. *Frontiers in Psychiatry*.

[47] Altshuler LL, Ventura J, van Gorp WG, Green MF, Theberge DC, Mintz J (2004). Neurocognitive function in clinically stable men with bipolar I disorder or schizophrenia and normal control subjects. *Biological Psychiatry*.

[48] Lang N, Siebner HR, Ernst D, Nitsche MA, Paulus W, Lemon RN (2004). Preconditioning with transcranial direct current stimulation sensitizes the motor cortex to rapid-rate transcranial magnetic stimulation and controls the direction of after-effects. *Biological Psychiatry*.

[49] Zhou H, Wang M, Xu T, Zhang X, Zhao X, Tang L (2024). Cognitive Remediation in Patients With Bipolar Disorder: A Randomized Trial by Sequential tDCS and Navigated rTMS Targeting the Primary Visual Cortex. *CNS Neuroscience & Therapeutics*.

[50] Dong X, Yan L, Huang L, Guan X, Dong C, Tao H (2018). Repetitive transcranial magnetic stimulation for the treatment of Alzheimer’s disease: A systematic review and meta-analysis of randomized controlled trials. *PloS One*.

[51] Iimori T, Nakajima S, Miyazaki T, Tarumi R, Ogyu K, Wada M (2019). Effectiveness of the prefrontal repetitive transcranial magnetic stimulation on cognitive profiles in depression, schizophrenia, and Alzheimer’s disease: A systematic review. *Progress in Neuro-psychopharmacology & Biological Psychiatry*.

[52] Li X, Qi G, Yu C, Lian G, Zheng H, Wu S (2021). Cortical plasticity is correlated with cognitive improvement in Alzheimer’s disease patients after rTMS treatment. *Brain Stimulation*.

[53] McGirr A, Karmani S, Arsappa R, Berlim MT, Thirthalli J, Muralidharan K (2016). Clinical efficacy and safety of repetitive transcranial magnetic stimulation in acute bipolar depression. *World Psychiatry: Official Journal of the World Psychiatric Association (WPA)*.

[54] Rapinesi C, Bersani FS, Kotzalidis GD, Imperatori C, Del Casale A, Di Pietro S (2015). Maintenance Deep Transcranial Magnetic Stimulation Sessions are Associated with Reduced Depressive Relapses in Patients with Unipolar or Bipolar Depression. *Frontiers in Neurology*.

