# Supplementary material for: Effects of Deep Transcranial Magnetic Stimulation on Cognitive Function in Bipolar Depression: A Randomized Controlled Trial Using the MATRICS Consensus Cognitive Battery
Source: Alpha Psychiatry. 2026 Feb 28;27(1):47409. doi: 10.31083/AP47409 (PMC12957972; doi:10.31083/AP47409)
Supplement: Supplementary file 1 [file 2757-8038-27-1-47409-s1.zip › Supplementary Material.docx]

**Supplementary Table 1. Baseline characteristics of completers and non-completers.**

|  | Completers(n=85) | Non-completers(n=15) | *t*/*χ*^2^ | *p* |
| --- | --- | --- | --- | --- |
| Age, mean ± SD, years | 38.44 ± 14.57 | 44.53 ± 17.08 | -1.46 | 0.15 |
| Sex, n (% Female) | 59 (69.4) | 9 (60.0) | 0.18 | 0.67^a^ |
| Marital status, n (% Married) | 52 (61.2) | 7 (46.7) | 1.11 | 0.29 |
| Occupational status, n (% Employed) | 35 (41.2) | 4 (26.7) | 1.13 | 0.29 |
| Educational level, mean ± SD, years | 12.48 ± 3.53 | 13.13 ± 3.31 | -0.66 | 0.51 |
| Age at illness onset, mean ± SD, years | 22.39 ± 7.97 | 21.20 ± 5.51 | 0.55 | 0.58 |
| Current episode duration, mean ± SD, months | 4.45 ± 5.51 | 6.40 ± 4.34 | -1.30 | 0.20 |
| Diagnosis subtype, n (%) |  |  |  |  |
| Bipolar Disorder Type I | 39 (45.9) | 7 (46.7) | 0.003 | 0.96 |
| Bipolar Disorder Type II | 46 (54.1) | 8 (53.3) | 0.003 | 0.96 |
| Current medication use, n (%) |  |  |  |  |
| First-line therapy | 76 (89.4) | 13 (86.7) |  | 0.67^b^ |
| Lithium | 53 (62.4) | 8 (53.3) | 0.44 | 0.51 |
| Valproate | 15 (17.6) | 4 (26.7) |  | 0.48^b^ |
| Lamotrigine | 26 (30.6) | 6 (40) | 0.18 | 0.67^a^ |
| Quetiapine | 35 (41.2) | 5 (33.3) | 0.33 | 0.57 |
| Benzodiazepine | 33 (38.8) | 6 (40) | 0.007 | 0.93 |
| HDRS-17 score, mean ± SD | 24.57 ± 6.16 | 22.93 ± 6.35 | 0.95 | 0.35 |
| YMRS score, mean ± SD | 3.58 ± 5.47 | 2.92 ± 2.64 | 0.46 | 0.65 |

HDRS-17, 17-item Hamilton Depression Rating Scale; YMRS, Young Mania Rating Scale.

^a^ The continuity correction was applied.
^b^ Fisher’s exact test was used.

**Supplementary Table 2. Comparison of cognitive scores between groups at baseline and week 4 (ITT analysis).**

|  | Active dTMS | | Sham dTMS | | *F*_Group_ | *F*_Time_ | *F*_Group*time_ | *η_p_*² | *η_p_*² | *η_p_*² | *p*_Time_ |
| --- | --- | --- | --- | --- | --- | --- | --- | --- | --- | --- | --- |
|  | Baseline | Week 4 | Baseline | Week 4 | (*p*_Group_) | (*p*_Time_) | (*p*_Group*time_) | (Group) | (Time) | (Group*time) | (FDR) |
| **Processing speed** | 36.65 ± 12.99 | 41.67 ± 11.81 | 36.89 ± 13.92 | 39.36 ± 13.31 | 0.16(0.69) | 22.72(<**0.001**) | 2.62(0.11) | 0.002 | 0.190 | 0.026 | <**0.001** |
| BACS-SC | 36.56 ± 13.84 | 39.87 ± 11.85 | 34.56 ± 11.10 | 37.02 ± 12.94 | 0.94(0.33) | 15.31(<**0.001**) | 0.35(0.56) | 0.010 | 0.136 | 0.004 | <**0.001** |
| Fluency | 44.14 ± 14.85 | 45.62 ± 11.54 | 45.89 ± 12.31 | 46.56 ± 12.65 | 0.29(0.59) | 1.11(0.295) | 0.16(0.69) | 0.003 | 0.011 | 0.002 | 0.317 |
| TMT-A | 39.63 ± 15.18 | 45.32 ± 13.87 | 39.83 ± 15.07 | 43.33 ± 12.86 | 0.11(0.74) | 15.61(<**0.001**) | 0.88(0.35) | 0.001 | 0.139 | 0.009 | <**0.001** |
| **Attention/Vigilance** |  |  |  |  |  |  |  |  |  |  |  |
| CPT-IP | 41.43 ± 8.98 | 45.38 ± 11.60 | 42.89 ± 11.75 | 46.31 ± 12.83 | 0.35(0.56) | 25.68(<**0.001**) | 0.17(0.71) | 0.004 | 0.209 | 0.001 | <**0.001** |
| **Working memory** |  |  |  |  |  |  |  |  |  |  |  |
| Spatial Span | 41.29 ± 8.20 | 45.98 ± 13.74 | 39.83 ± 9.88 | 42.04 ±9.82 | 1.17(0.28) | 9.00(**0.003**) | 0.48(0.49) | 0.012 | 0.085 | 0.005 | **0.004** |
| **Verbal learning** |  |  |  |  |  |  |  |  |  |  |  |
| HVLT-R | 38.87 ± 11.39 | 44.02 ± 13.68 | 40.11 ±13.46 | 46.67 ± 13.50 | 0.60(0.44) | 28.46(<**0.001**) | 0.24(0.62) | 0.006 | 0.227 | 0.003 | <**0.001** |
| **Visual learning** |  |  |  |  |  |  |  |  |  |  |  |
| BVMT-R | 46.73 ± 13.65 | 48.75 ± 14.48 | 51.44 ± 11.70 | 52.64 ± 12.56 | 2.66(0.11) | 2.75(0.101) | 0.383(0.54) | 0.027 | 0.028 | 0.004 | 0.118 |
| **Reasoning and problem solving** |  |  |  |  |  |  |  |  |  |  |  |
| NAB Mazes | 40.30 ± 10.88 | 45.73 ± 12.69 | 41.00 ± 7.75 | 43.69 ± 8.09 | 0.17(0.68) | 18.32(<**0.001**) | 2.77(0.10) | 0.002 | 0.159 | 0.028 | <**0.001** |
| **Social cognition** |  |  |  |  |  |  |  |  |  |  |  |
| MSCEIT-ME | 32.91 ± 5.64 | 35.94 ± 6.04 | 35.17 ± 7.67 | 37.88 ± 8.33 | 2.66(0.11) | 21.73(<**0.001**) | 0.46(0.50) | 0.027 | 0.183 | 0.005 | <**0.001** |
| **Mean composite score** | 33.30 ± 10.91 | 39.67 ± 13.73 | 35.67 ± 11.72 | 40.36 ± 11.97 | 0.46(0.50) | 49.18(**< 0.001**) | 1.12(0.29) | 0.005 | 0.336 | 0.011 | <**0.001** |

dTMS, deep transcranial magnetic stimulation; MCCB, MATRICS Consensus Cognitive Battery; **BACS-SC,** Brief Assessment of Cognition in Schizophrenia**–**Symbol Coding; **Fluency,** Category Fluency (Animal Naming); **TMT-A,** Trail Making Test, Part A; **CPT-IP,** Continuous Performance Test**–Identical Pairs**; **HVLT-R,** Hopkins Verbal Learning Test–Revised; **BVMT-R,** Brief Visuospatial Memory Test–Revised; **NAB-Mazes,** Neuropsychological Assessment Battery**–**Mazes; **MSCEIT-ME,** Mayer-Salovey-Caruso Emotional Intelligence Test–Managing Emotions; ITT, intention-to-treat; FDR, false discovery rate. Bold values indicate *p* < 0.05.

**Supplementary Table 3. Comparison of cognitive scores between groups at baseline and week 4 (PP analysis).**

|  | Active dTMS | | Sham dTMS | | *F*_Group_ | *F*_Time_ | *F*_Group*time_ | *η_p_*² | *η_p_*² | *η_p_*² | *p*_Time_ |
| --- | --- | --- | --- | --- | --- | --- | --- | --- | --- | --- | --- |
|  | Baseline | Week 4 | Baseline | Week 4 | (*p*_Group_) | (*p*_Time_) | (*p*_Group*time_) | (Group) | (Time) | (Group*time) | (FDR) |
| **Processing speed** | 35.85 ± 12.94 | 41.70 ± 11.75 | 35.23 ± 13.26 | 38.20 ± 13.21 | 0.57(0.45) | 23.76(**< 0.001**) | 2.54(0.12) | 0.007 | 0.225 | 0.030 | **<0.001** |
| BACS-SC | 35.57 ± 12.95 | 39.44 ± 11.33 | 32.90 ± 10.43 | 35.77 ± 12.10 | 1.52(0.22) | 17.52(**< 0.001**) | 0.39(0.54) | 0.018 | 0.176 | 0.005 | **<0.001** |
| Fluency | 44.11 ± 15.27 | 46.50 ± 11.01 | 45.73 ± 12.22 | 45.70 ± 13.26 | 0.02(0.88) | 1.16(0.285) | 1.23(0.27) | 0.001 | 0.014 | 0.015 | 0.313 |
| TMT-A | 38.85 ± 14.53 | 45.17 ± 13.92 | 37.93 ± 13.97 | 42.07 ± 12.01 | 0.49(0.49) | 16.98(**<0.001**) | 0.74(0.39) | 0.006 | 0.172 | 0.009 | **<0.001** |
| **Attention/Vigilance** |  |  |  |  |  |  |  |  |  |  |  |
| CPT-IP | 41.04 ± 8.70 | 45.65 ± 11.11 | 42.53 ± 10.72 | 46.63 ± 12.25 | 0.30(0.58) | 26.96(<**0.001**) | 0.09(0.76) | 0.004 | 0.247 | 0.001 | **<0.001** |
| **Working memory** |  |  |  |  |  |  |  |  |  |  |  |
| Spatial Span | 41.54 ± 7.92 | 45.85 ± 14.40 | 38.97 ± 9.42 | 41.73 ± 9.91 | 2.30(0.13) | 9.09(**0.003**) | 0.44(0.51) | 0.027 | 0.100 | 0.005 | **0.005** |
| **Verbal learning** |  |  |  |  |  |  |  |  |  |  |  |
| HVLT-R | 38.74 ± 10.31 | 45.09 ± 13.61 | 38.80 ± 12.33 | 46.67 ± 13.98 | 0.10(0.75) | 30.09(**<0.001**) | 0.34(0.56) | 0.001 | 0.268 | 0.004 | **<0.001** |
| **Visual learning** |  |  |  |  |  |  |  |  |  |  |  |
| BVMT-R | 45.85 ± 13.68 | 48.91 ± 14.20 | 51.27 ± 12.09 | 52.70 ± 12.17 | 2.88(0.09) | 2.73(0.102) | 0.36(0.55) | 0.034 | 0.032 | 0.004 | 0.118 |
| **Reasoning and problem solving** |  |  |  |  |  |  |  |  |  |  |  |
| NAB Mazes | 40.20 ± 10.72 | 46.54 ± 12.77 | 40.90 ± 7.69 | 43.77 ± 8.38 | 0.23(0.64) | 18.96(**<0.001**) | 2.69(0.11) | 0.003 | 0.188 | 0.032 | **<0.001** |
| **Social cognition** |  |  |  |  |  |  |  |  |  |  |  |
| MSCEIT-ME | 33.17 ± 5.42 | 36.47 ± 6.16 | 34.97 ± 7.80 | 37.50 ± 6.46 | 1.18(0.28) | 22.62(**<0.001**) | 0.39(0.54) | 0.014 | 0.216 | 0.005 | **<0.001** |
| **Mean composite score** | 32.98 ± 10.08 | 40.41 ± 14.06 | 34.60 ± 9.31 | 40.23 ± 11.68 | 0.08(0.77) | 54.57(**< 0.001**) | 1.03(0.31) | 0.001 | 0.400 | 0.012 | **<0.001** |

dTMS, deep transcranial magnetic stimulation; MCCB, MATRICS Consensus Cognitive Battery; BACS, **BACS-SC,** Brief Assessment of Cognition in Schizophrenia**–**Symbol Coding; **Fluency,** Category Fluency (Animal Naming); **TMT-A,** Trail Making Test, Part A; **CPT-IP,** Continuous Performance Test**–Identical Pairs**; **HVLT-R,** Hopkins Verbal Learning Test–Revised; **BVMT-R,** Brief Visuospatial Memory Test–Revised; **NAB-Mazes,** Neuropsychological Assessment Battery**–**Mazes; **MSCEIT-ME,** Mayer-Salovey-Caruso Emotional Intelligence Test–Managing Emotions; PP, per-protocol; FDR, false discovery rate. Bold values indicate *p* < 0.05.

**Supplementary Table 4. Comparison of cognitive scores between groups at baseline and week 4 (Multiple Imputation Analysis).**

|  | Active dTMS | | Sham dTMS | | *F*_Group_ | *F*_Time_ | *F*_Group*time_ | *η_p_*² | *η_p_*² | *η_p_*² | *p*_Time_ |
| --- | --- | --- | --- | --- | --- | --- | --- | --- | --- | --- | --- |
|  | Baseline | Week 4 | Baseline | Week 4 | (*p*_Group_) | (*p*_Time_) | (*p*_Group*time_) | (Group) | (Time) | (Group*time) | (FDR) |
| **Processing speed** | 36.65 ± 12.99 | 41.56 ± 11.73 | 36.89 ± 13.92 | 39.76 ± 13.09 | 0.10(0.76) | 21.34**(<0.001**) | 1.47(0.23) | 0.001 | 0.180 | 0.015 | **<0.001** |
| BACS-SC | 36.56 ± 13.84 | 40.07 ± 12.60 | 34.56 ± 11.10 | 37.01 ± 11.99 | 0.99(0.32) | 16.13**(<0.001)** | 0.50(0.48) | 0.010 | 0.143 | 0.005 | **<0.001** |
| Fluency | 44.14 ± 14.85 | 45.89 ± 11.27 | 45.89 ± 12.31 | 46.31 ± 12.37 | 0.19(0.67) | 1.22(0.27) | 0.45(0.50) | 0.002 | 0.012 | 0.005 | 0.307 |
| TMT-A | 39.63 ± 15.18 | 45.25 ± 13.99 | 39.83 ± 15.07 | 43.29 ± 12.98 | 0.10(0.75) | 15.54**(<0.001)** | 0.88(0.35) | 0.001 | 0.138 | 0.009 | **<0.001** |
| **Attention/Vigilance** |  |  |  |  |  |  |  |  |  |  |  |
| CPT-IP | 41.43 ± 8.98 | 45.94 ± 10.96 | 42.89 ± 11.75 | 46.60 ± 11.86 | 0.28(0.60) | 24.02**(<0.001**) | 0.23(0.63) | 0.003 | 0.198 | 0.002 | **<0.001** |
| **Working memory** |  |  |  |  |  |  |  |  |  |  |  |
| Spatial Span | 41.29 ± 8.20 | 45.45 ± 14.81 | 39.83 ± 9.88 | 41.62 ± 9.57 | 1.77(0.19) | 6.31**(0.014)** | 1.00(0.32) | 0.018 | 0.061 | 0.010 | **0.017** |
| **Verbal learning** |  |  |  |  |  |  |  |  |  |  |  |
| HVLT-R | 38.87 ± 11.39 | 44.99 ± 13.39 | 40.11 ±13.46 | 46.26 ± 13.46 | 0.32(0.57) | 21.12**(<0.001)** | 0.01(0.92) | 0.003 | 0.179 | <0.001 | **<0.001** |
| **Visual learning** |  |  |  |  |  |  |  |  |  |  |  |
| BVMT-R | 46.73 ± 13.65 | 50.95 ± 17.81 | 51.44 ± 11.70 | 50.56 ± 13.59 | 0.69(0.41) | 1.01(0.32) | 2.37(0.13) | 0.007 | 0.010 | 0.024 | 0.327 |
| **Reasoning and problem solving** |  |  |  |  |  |  |  |  |  |  |  |
| NAB Mazes | 40.30 ± 10.88 | 46.32 ± 12.52 | 41.00 ± 7.75 | 44.11 ± 8.82 | 0.17(0.70) | 21.27**(<0.001)** | 2.15(0.15) | 0.002 | 0.180 | 0.022 | **<0.001** |
| **Social cognition** |  |  |  |  |  |  |  |  |  |  |  |
| MSCEIT-ME | 32.91 ± 5.64 | 36.56 ± 6.13 | 35.17 ± 7.67 | 37.76 ± 6.68 | 2.33(0.13) | 24.34**(<0.001)** | 0.69(0.41) | 0.023 | 0.201 | 0.007 | **<0.001** |
| **Mean composite score** | 33.30 ± 10.91 | 40.56 ± 13.17 | 35.67 ± 11.72 | 40.11 ± 10.92 | 0.20(0.66) | 37.97**(<0.001)** | 2.20(0.14) | 0.002 | 0.281 | 0.022 | **<0.001** |

dTMS, deep transcranial magnetic stimulation; MCCB, MATRICS Consensus Cognitive Battery; **BACS-SC,** Brief Assessment of Cognition in Schizophrenia**–**Symbol Coding; **Fluency,** Category Fluency (Animal Naming); **TMT-A,** Trail Making Test, Part A; **CPT-IP,** Continuous Performance Test**–Identical Pairs**; **HVLT-R,** Hopkins Verbal Learning Test–Revised; **BVMT-R,** Brief Visuospatial Memory Test–Revised; **NAB-Mazes,** Neuropsychological Assessment Battery**–**Mazes; **MSCEIT-ME,** Mayer-Salovey-Caruso Emotional Intelligence Test–Managing Emotions; FDR, false discovery rate. Bold values indicate *p* < 0.05.

**Supplementary Table 5. Comparison of cognitive scores between groups at baseline and week 4 (Worst-Case Scenario Analysis).**

|  | Active dTMS | | Sham dTMS | | *F*_Group_ | *F*_Time_ | *F*_Group*time_ | *η_p_*² | *η_p_*² | *η_p_*² | *p*_Time_ |
| --- | --- | --- | --- | --- | --- | --- | --- | --- | --- | --- | --- |
|  | Baseline | Week 4 | Baseline | Week 4 | (*p*_Group_) | (*p*_Time_) | (*p*_Group*time_) | (Group) | (Time) | (Group*time) | (FDR) |
| **Processing speed** | 36.65 ± 12.99 | 41.05 ± 11.92 | 36.89 ± 13.92 | 38.47 ± 13.06 | 0.21(0.65) | 12.54**(<0.001)** | 2.79(0.10) | 0.002 | 0.114 | 0.028 | **<0.001** |
| BACS-SC | 36.56 ± 13.84 | 38.95 ± 12.36 | 34.56 ± 11.10 | 36.08 ± 11.87 | 0.95(0.33) | 6.49**(0.012)** | 0.32(0.57) | 0.010 | 0.063 | 0.003 | **0.016** |
| Fluency | 44.14 ± 14.85 | 45.10 ± 11.57 | 45.89 ± 12.31 | 44.84 ± 13.32 | 0.09(0.77) | 0.01(0.96) | 1.05(0.31) | 0.001 | <0.001 | 0.011 | 0.964 |
| TMT-A | 39.63 ± 15.18 | 44.01± 14.83 | 39.83 ± 15.07 | 42.11 ± 14.01 | 0.09(0.76) | 8.07**(0.005)** | 0.80(0.37) | 0.001 | 0.077 | 0.008 | **0.007** |
| **Attention/Vigilance** |  |  |  |  |  |  |  |  |  |  |  |
| CPT-IP | 41.43 ± 8.98 | 44.82 ± 10.78 | 42.89 ± 11.75 | 45.52 ± 12.27 | 0.29(0.60) | 14.89**(<0.001)** | 0.23(0.63) | 0.003 | 0.133 | 0.002 | **<0.001** |
| **Working memory** |  |  |  |  |  |  |  |  |  |  |  |
| Spatial Span | 41.29 ± 8.20 | 44.25 ± 14.13 | 39.83 ± 9.88 | 41.11 ± 9.88 | 1.31(0.26) | 4.07**(0.046)** | 0.64(0.43) | 0.013 | 0.040 | 0.007 | 0.057 |
| **Verbal learning** |  |  |  |  |  |  |  |  |  |  |  |
| HVLT-R | 38.87 ± 11.39 | 43.34 ± 13.93 | 40.11 ±13.46 | 45.66 ± 13.69 | 0.58(0.45) | 17.24**(<0.001)** | 0.20(0.66) | 0.006 | 0.151 | 0.002 | **<0.001** |
| **Visual learning** |  |  |  |  |  |  |  |  |  |  |  |
| BVMT-R | 46.73 ± 13.65 | 48.58 ± 13.86 | 51.44 ± 11.70 | 51.99 ± 11.67 | 2.77(0.10) | 1.04(0.31) | 0.30(0.58) | 0.028 | 0.011 | 0.003 | 0.325 |
| **Reasoning and problem solving** |  |  |  |  |  |  |  |  |  |  |  |
| NAB Mazes | 40.30 ± 10.88 | 45.34 ± 12.88 | 41.00 ± 7.75 | 42.74 ± 8.37 | 0.23(0.64) | 12.90**(<0.001)** | 3.06(0.08) | 0.002 | 0.117 | 0.031 | **<0.001** |
| **Social cognition** |  |  |  |  |  |  |  |  |  |  |  |
| MSCEIT-ME | 32.91 ± 5.64 | 35.02 ± 6.79 | 35.17 ± 7.67 | 36.50 ± 6.70 | 2.42(0.12) | 8.29**(0.005)** | 0.41(0.52) | 0.024 | 0.079 | 0.004 | **0.007** |
| **Mean composite score** | 33.30 ± 10.91 | 38.74 ± 14.02 | 35.67 ± 11.72 | 39.26 ± 11.61 | 0.41(0.53) | 25.23**(<0.001)** | 1.06(0.31) | 0.004 | 0.206 | 0.011 | **<0.001** |

dTMS, deep transcranial magnetic stimulation; MCCB, MATRICS Consensus Cognitive Battery; **BACS-SC,** Brief Assessment of Cognition in Schizophrenia**–**Symbol Coding; **Fluency,** Category Fluency (Animal Naming); **TMT-A,** Trail Making Test, Part A; **CPT-IP,** Continuous Performance Test**–Identical Pairs**; **HVLT-R,** Hopkins Verbal Learning Test–Revised; **BVMT-R,** Brief Visuospatial Memory Test–Revised; **NAB-Mazes,** Neuropsychological Assessment Battery**–**Mazes; **MSCEIT-ME,** Mayer-Salovey-Caruso Emotional Intelligence Test–Managing Emotions; FDR, false discovery rate. Bold values indicate *p* < 0.05.
